# 

**Published:** 1918-06-22

**Authors:** 


Rates of Subscription: Twelve months, 6/6; Six months, 4/-; three months, 2/-, post free to any address in the United Kingdom. Abroad, Twelve months, 8/8; Six months, 5/-; Three months, 2/6, post free. THE HOSPITAL "Index" to Volume LXI October 1917 to March 1918, is now ready, price 3½d. per copy, post free. Address The Manager, The Hospital, "The Hospital" Building, 28/29 Southampton Street, Strand, London, W.C. 2.

**DO SO NOW**

After Monday, June 24th, you will not be able to get The Hospital unless you give your Newsagent a definite order.

**DO SO NOW**

### HUNS IN HOTELS.

### A CALL TO EXCLUDE ALL ALIEN ENEMIES.

The proprietor of an important Midland hotel makes a valuable suggestion to the National Party in reference to the boycott of Germans after the war. He asks everyone in control of hotels in Great Britain not to house any alien enemy. "This," he writes, "would make it difficult for these brutes to conduct their business as in pre-war times, when they flooded our hotels and dumped their goods everywhere. I am sorry to say that there are a great number of Englishmen who will buy goods from Germany if they can get them cheaper than British-made articles. I shall bar alien enemies from any hotel with which I am associated." General Page Croft, M.P., is cordially in sympathy with the suggestion, and he recommends all who agree with the idea to write to 22 King Street, London, S.W. 1, for a National Boycott Pledge.—*The Director of Propaganda, National Party, 22 King Street, S.W.* 1.

**DO SO NOW**

After Monday, June 24th, you will not be able to get The Hospital unless you give your Newsagent a definite order.

**DO SO NOW**

### Matrons' and Sisters' Appointments.

Babies' Castle, Hawkhurst.—Miss Elizabeth Gillies has been appointed assistant matron. She was trained at the Glasgow Royal Infirmary, later being sister there, and has been matron at Bromyard Cottage Hospital.

Crumpsall Infirmary, Manchester. — Miss Elizabeth Eleanor Sealey has been appointed night superintendent. She was trained at Crumpsall Infirmary, where she has also been ward sister. She holds the certificate of the Central Midwives Board.

Edenbridge and Great Central War Hospitals, Carlisle. —Miss E. Unsworth has been appointed matron. She was trained at University College Hospital, and has since been matron of Newton Abbot V.A. Hospital and Regent's Park Red Cross Hospital, Southampton. Miss Catherine MacQuarrie has been appointed night sister. She was trained at Bolton Infirmary and City Hospital, Edinburgh; she has since been a Queen's nurse at Edinburgh and sister at Newton Abbot V.A. Hospital. Miss Edith Fletcher has been appointed sister in charge of the Great Central Section. Trained at Guy's Hospital, since sister at Graylingwell War Hospital and assistant matron of Fusehill War Hospital. The following have been appointed sisters: Miss E. Collins, trained at City of London Infirmary, later theatre sister; Miss Mary MacDowell, trained at St. Pancras Infirmary and North Eastern Fever Hospital, since sister at Newton Abbot V.A. Hospital and at Regent's Park Red Cross Hospital, Southampton; Miss Effie Saunders, trained at Aberdeen Royal Infirmary, since sister at Newton Abbot and Regent's Park, Southampton; Miss Bertha Poole, trained at Guy's Hospital, since staff nurse at Graylingwell War Hospital, sister at Berkeley Square Officers' Hospital; Miss Margaret Macdougall, trained at Victoria Hospital, Accrington, later sister; Miss A. Armstrong, trained at St. James' Infirmary, Balham, since theatre and ward sister at Darlington General Hospital and sister at Derbyshire Royal Infirmary.

Maternity and Infants' Hospital, Rochdale.—Miss Jessie Woolford has been appointed night sister. She was trained at the Union Infirmary, Blackburn; has been staff nurse at the Bradford War Hospital, sister at Stepping Hill Military Hospital, sister in charge of an ambulance room in a shell factory, and latterly has been ward sister at the Union Infirmary, Blackburn. She holds the C.M.B. certificate.

**NOTE.—Particulars of Appointments for insertion in this column will be welcomed.**

### Hot Weather Possibilities.

LOCAL authorities are being warned to take precautions against the possibility of diseases this summer. The Camberwell Medical Officer of Health, for instance, directs attention to the possibility of the spread of malaria through the agency of mosquitoes. The breeding-places of these insects are pools of stagnant water and uncovered and disused cisterns, over which the sanitary inspectors have been asked to be vigilant. Local authorities are urging for a double milk delivery, pointing out that one delivery a day is not enough in the hot weather, when milk so quickly turns sour.

### Annual Meetings.

ROYAL NATIONAL PENSION FUND FOR NURSES.—Thursday, June 27, 4 P.M., at the Society of Arts, John Street, Adelphi.

